# Dengue Vaccine: Considerations before Rollout in Colombia

**DOI:** 10.1371/journal.pntd.0004653

**Published:** 2016-06-09

**Authors:** Christian Julian Villabona-Arenas, Raquel Elvira Ocazionez Jimenez, Cinthy Lorena Jimenez Silva

**Affiliations:** 1 UMI233, Institut de Recherche pour le Développement (IRD), Université de Montpellier, Montpellier, France; 2 Laboratoire d'Informatique, de Robotique et de Microélectronique de Montpellier (LIRMM), Institut de Biologie Computationnelle (IBC), Université de Montpellier, Montpellier, France; 3 Laboratorio de Arbovirus, Centro de Investigaciones en Enfermedades Tropicales (CINTROP), Universidad Industrial de Santander, Santander, Colombia; Duke-NUS GMS, SINGAPORE

Dengue has become an increasing public health concern in tropical and subtropical countries with worsening societal and financial burdens. Dengue causes considerable suffering and loss of productivity despite the typical, relatively short, weeklong duration of illness. Furthermore, people keep dying from dengue in the absence of early clinical recognition of severe infection and adequate supportive care. The World Health Organization estimates that 50–100 million dengue infections occur annually. However, estimates based on cartographic modelling approaches suggest that up to 390 million dengue infections occur annually (95% CI 284–528) [1]. The Pan American Health Organization (PAHO) reported in the region of the Americas a total of 1,181 deaths in 2015. Dengue costs are substantial because of the cost of hospital care and the loss in earnings by incapacitation or premature death [2]. Estimates are probably conservative because most studies do not include budgets directed to vector control, loss in tourism, and other costs.

A vaccine to control dengue is urgently needed. Currently, there are various candidates in different stages of clinical development. The Sanofi Pasteur tetravalent chimeric yellow-fever dengue vaccine, named CYD-TDV, finished Phase III clinical studies in Latin America; this trial enrolled a total of 20,869 children aged 9 to 16 years from endemic areas in five countries [3]. A comparable trial was first completed in endemic areas of Southeast Asian countries [4]. Both studies assessed the efficacy of CYD-TDV after three doses in preventing symptomatic virologically confirmed cases. The vaccine has been qualified as efficacious by the Latin American study’s authors with rates of efficacy for symptomatic dengue during the first 25 months (intention-to-treat) being 64.7% (95% CI 58.7–69.8) and rates of efficacy against serotypes DEN-1, DEN-2, DEN-3, and DEN-4 being 50%, 42%, 74%, and 78%, respectively [3].

A total of nine locations from Colombia enrolled in the CYD-TDV trial since June 2011 [3,5]. The per capita estimated economic dengue burden for Colombia’s health care system has been estimated at US$52.80, US$235.80, and US$1,512.20 per case for ambulatory cases, hospitalized cases, and severe hospitalized cases, respectively [2]. During 2015, 94,916 clinical cases were reported from Colombia to PAHO, including 1,360 severe cases and 72 deaths. Cartographic approaches led to a yearly average of apparent dengue infections of 1,073,891 (95% CI 783,699–1,465,285) [1].

Santander is one of the 32 states of Colombia. Bucaramanga is its capital and, together with three nearby municipalities, constitutes the seventh-largest metropolitan area of the country; Bucaramanga participated in the CYD-TDV trial [3,5]. *Aedes aegypti* thrives in Santander’s tropical climate and, consequently, 78% of the municipalities are considered endemic for dengue [6]; the state accounted for the highest number of cases reported (13.05%) in the period between 1990–2010. Santander occupies the fourth position in the national gross domestic product ranking (GDP) and, together with the Norte de Santander State (the region north of Santander), serves as a point of transit with the adjacent country of Venezuela. Given these factors, substantial morbidity due to dengue and cross-border flow of dengue-infected persons is expected to continue.

DEN-1, -2, and -3 were documented for the first time in Colombia during the 1970s, and DEN-4 was introduced in 1982 [6]. The prevalence of serotypes has changed over time, and, to date, multiple serotypes have been reported in 30 states. We previously studied the temporal patterns of dengue serotypes circulation in the region of Santander. DEN-3 was reintroduced in 2001 and became the predominant serotype until 2004 (74.7% versus 3.0% DEN-1, 14.1% DEN-2, and 8.1% DEN-4) [7]; then, during the period between 2005–2008, DEN-1 and -2 increased in frequency (31.5% DEN-1; 22.8% DEN-2, 34.9% DEN-3, and 10.7% DEN-4) [8–11]. Data from 2009–2013 are not available, and during the period 2014–2015, we found that DEN-1 and -2 were the most prevalent (38.2% DEN-1, 36.0% DEN-2, 16.8% DEN-3, and 9.0% DEN-4) (Fig 1); DEN-4 has been the less prevalent serotype regardless of the time period. The study’s authors of the CYD-TDV trial (2011–2014) documented that DEN-3 (40.1%) and DEN-1 (34.7%) were the most common serotypes for Colombia, followed by DEN-2 (19.8%) and DEN-4 (5.4%) [3]. Comprehensive data on serotype prevalence or change over time for the country and other regions are very limited [12].

**Fig 1:**
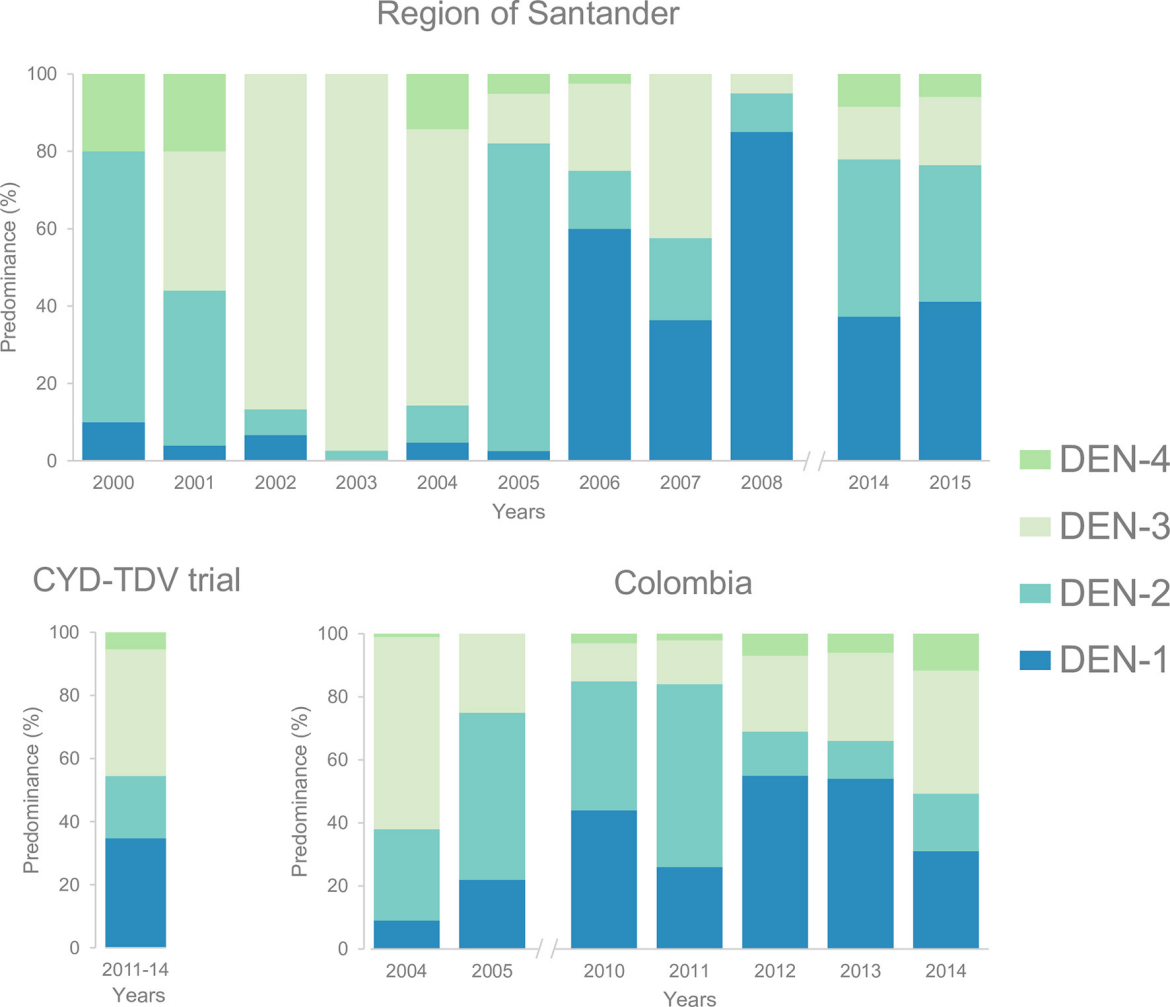
Relative predominance of dengue serotypes in Colombia. Santander data correspond to aggregated observations in the metropolitan area of Bucaramanga (State of Santander) and the province of Ocaña (State of Norte de Santander) reported by the Grupo de Investigación y Enfermedades Tropicales (CINTROP) from Industrial University of Santander (UIS) [8–11]. The CYD-TDV data correspond to the country-specific infecting serotype of virologically confirmed dengue in the intention-to-treat group [3]. Data for the whole country come from the National Institute of Health (Colombia).

Given limited countrywide surveillance, there is a lack of definitive evidence to characterize the links between serotype and incidence or severity of disease [12]. However, when DEN-2 is the most predominant serotype in the region of Santander, we observed an increase in the proportion of clinical cases that are diagnosed as secondary infections (51.6% versus 18.3%, 21.8%, and 8.3% when DEN-1, -3, and -4 are more common, respectively), which is a risk factor for severe disease [9,11]. In addition, we have isolated DEN-1 and -2 more often from severe cases (proportion of patients suffering severe cases in respect to serotype: 21% DEN-1, 28% DEN-2, 11% DEN-3, and 0% DEN-4), and those were the only serotypes associated with fatalities (one and four occurrences for DEN-1 and DEN-2, respectively) [9,11]. In 2014 in Colombia, most dengue-related fatalities were associated with DEN-2 (36% versus 19% DEN-1, 26% DEN-3, and 19% DEN-4) even though it was the third most common serotype (30% DEN-1, 18% DEN-2, 40% DEN-3, and 12% DEN-4) [13].

An increase in the relative incidence of dengue in children less than 15 years of age has been documented over time in Colombia; from 2004 to 2010, the highest reported incidence was in the ≤4 years and 5–14 years age groups [6,12]. In 2014, 23.6% of cases affected children 9 years of age and under, 16.8% of cases affected children in the 10–14 range, and 12.7% of cases affected adolescents and young adults in the 15–19 range; the remaining cases were distributed in older age classes in decreasing order [13]. Since 2003, there has been a significant increase in deaths in individuals aged <14 years and >45 years [12]. An increase in the loss of income over consecutive years has been attributed to premature death, presumably as a result of the lower average age of the patients who died [2].

Dengue in Colombia is a serious health public problem, and it will be a great benefit to have a vaccine. Nonetheless, policymakers should take into account the aforementioned local epidemiological settings before vaccine rollout. National data of temporal serotype predominance are aggregated measures from dengue-endemic areas with different characteristics, but serotype prevalence changes regularly over the years and locations. For example, we reported DEN-1 and DEN-2 as the most prevalent serotypes in 2014 in Santander, whereas DEN-1 and -3 were the most prevalent serotypes in the country according to the Colombian Institute of Health. Given the serotype-specific vaccine efficacies, protection will vary according to how prevalent serotypes are at each location; moreover, CYD-TDV Phase III trial results for DEN-1 and -2 fall short of the levels of protection required for a standalone vaccine intervention. High prevalence of these serotypes in Colombia raises concern about the long-term protection due to reduced efficacy and the observed local outcome of DEN-2 infections.

Children younger than 9 years of age were not included in the Latin American CYD-TDV trial; inferences for this age group come from the CYD-TDV in Asia, where the vaccine efficacy was far lower when compared to older children. For example, the highest relative risk for hospitalization for children between the ages of 2 and 5 years was 4.7 (95% CI 1.1–313.8), while for children between the ages of 6 and 11 years it was 0.63 (95% CI 0.22–1.83) [4]. Although studies regarding long-term safety and efficacy are being conducted at the present time, observations done after year three of the trials in Asia suggest an overall trend to increased risk in the vaccine group for participants younger than 9 years of age [4,5]. These findings highlight the need for monitoring the local risk–benefit profile of CYD-TDV in the youngest age groups.

A reduction of dengue mortality by half and morbidity by 25% would present a substantial public health benefit that would support vaccine introduction and match WHO goals [5]. Nonetheless, local changing patterns of dengue prevalence should accompany vaccine efficacy findings if trials on pharmacovigilance are set in place. We advise longer observation times to conclusively rule out increased disease severity associated with vaccination due to antibody-dependent enhancement of infection phenomenon and detailed insights on the immunological background of the Colombian population (e.g., detection of serotype-specific antibodies) to assess the impact of age and serostatus on disease severity and the impact of vaccination on infection and disease [16]. This will help inform a vaccination plan and may contribute to understanding the interactions that lead to changes in serotype prevalence. Moreover, the measurement of infection, not just disease, should be considered in dengue vaccine trials given that asymptomatic infection patients are also infectious to mosquitoes [17].

There is a global call for the need for research on improved integrated surveillance and the integration of vector control with vaccine introduction even though there is limited information about the efficacy of vector control strategies [14]. Conventional means of controlling mosquito populations have not been successful in Colombia, and this favoured the introduction and autochthonous transmission of Chikungunya virus (75,000 cases confirmed up to December 2014) and Zika virus (more than 20,000 cases confirmed up to February 2016) in 2014 and 2015, respectively (https://www.minsalud.gov.co/). Given the pandemic expansion of multiple arboviruses, efforts should be placed on molecular detection not only for disease management but also for adequate vaccine efficacy assessment. Chikungunya virus infections are similar to several flavivirus infections, and serological cross-reactivity is strong between Zika and dengue viruses [15]. Consequently, we consider it important to ensure a robust system of surveillance and community awareness and involvement toward mosquito control before and after vaccine introduction.

In the absence of a comprehensive supplementary vector control strategy, it is difficult to anticipate how the deployment of tetravalent vaccines that are not equally effective against all four serotypes will affect local dengue morbidity. Likewise, there are limited studies on any reasonable immunogenic interaction resulting from yellow fever immunization programmes and the Zika virus outbreak. A tetravalent vaccine effective against all four dengue serotypes may be the best approach to effectively control the disease. In the light of the actual results, some research gaps, and local epidemiological features, we stress the need for making careful decisions before the introduction of dengue vaccination in Colombia. We suggest vaccine introduction in selected communities (e.g., areas of highest incidence) before embarking in a national rollout; this stepwise introduction should include simultaneous evaluation of clinical outcome, age-specific seroprevalence, serotype prevalence, and community mobilization toward control. There are also local advocates on the necessity of genotypic detailed surveillance at the country level before immunization [18]. Health authorities should assess the adequate size of communities in such a preliminary rollout and perform comparable documented vector control activities in areas with and without vaccination. Importantly, authorities should guarantee the country’s development of surveillance for adverse advents or pharmacovigilance, laboratory quality control programmes, and the training of health care providers in dengue case management so that specific instances of the disease are documented consistently.

## Ethics Statement

Samples from 2000–2002, 2005–2008, and 2014–2015 were febrile cases reported routinely to the dengue surveillance programme from the local Secretary of Health. Informed consent was not obtained from these patients; the data were analysed anonymously. The Grupo de Epidemiología Clínica at Universidad Industrial de Santander (UIS) conducted a cross-sectional study during 2003–2004 in which patients gave written informed consent and sera were provided for virus isolation to the Centro de Investigaciones en Enfermedades Tropicales (CINTROP) from UIS. The Ethic board at UIS (Comité de Ética de Investigación Científica—CIENCI) approved the research.
